# Inhaled corticosteroids and FEV_1_ decline in chronic obstructive pulmonary disease: a systematic review

**DOI:** 10.1186/s12931-019-1249-x

**Published:** 2019-12-04

**Authors:** Hannah R. Whittaker, Debbie Jarvis, Mohamed R. Sheikh, Steven J. Kiddle, Jennifer K. Quint

**Affiliations:** 1 National Heart and Lung Institute, Imperial College London, Emmanuel Kaye Building, 1b Manresa Road, London, SW3 6LR UK; 2 MRC Biostatistics, University of Cambridge, Cambridge, UK

**Keywords:** Lung function, COPD, Inhaled corticosteroids, Review

## Abstract

Rate of FEV_1_ decline in COPD is heterogeneous and the extent to which inhaled corticosteroids (ICS) influence the rate of decline is unclear. The majority of previous reviews have investigated specific ICS and non-ICS inhalers and have consisted of randomised control trials (RCTs), which have specific inclusion and exclusion criteria and short follow up times. We aimed to investigate the association between change in FEV_1_ and ICS-containing medications in COPD patients over longer follow up times.

MEDLINE and EMBASE were searched and literature comparing change in FEV_1_ in COPD patients taking ICS-containing medications with patients taking non-ICS-containing medications were identified. Titles, abstract, and full texts were screened and information extracted using the PICO checklist. Risk of bias was assessed using the Cochrane Risk of Bias tool and a descriptive synthesis of the literature was carried out due to high heterogeneity of included studies.

Seventeen studies met our inclusion criteria. We found that the difference in change in FEV_1_ in people using ICS and non-ICS containing medications depended on the study follow-up time. Shorter follow-up studies (1 year or less) were more likely to report an increase in FEV_1_ from baseline in both patients on ICS and in patients on non-ICS-containing medications, with the majority of these studies showing a greater increase in FEV_1_ in patients on ICS-containing medications. Longer follow-up studies (greater than 1 year) were more likely to report a decline in FEV_1_ from baseline in patients on ICS and in patients on non-ICS containing medications but rates of FEV_1_ decline were similar.

Further studies are needed to better understand changes in FEV_1_ when ICS-containing medications are prescribed and to determine whether ICS-containing medications influence rate of decline in FEV_1_ in the long term. Results from inclusive trials and observational patient cohorts may provide information more generalisable to a population of COPD patients.

## Background

Chronic obstructive pulmonary disease (COPD) is a progressive disease characterized by the chronic obstruction of airflow in the airways and lungs. Evidence based clinical NICE guidelines recommend the use of inhaled bronchodilators such as long-acting beta-2 adrenergic receptor agonists (LABA) or long-acting muscarinic-receptor antagonists (LAMA) for COPD maintenance therapy [1, 2]. Currently, the addition of inhaled corticosteroids (ICS) is reserved for those who remain breathless or exacerbate despite taking short-acting bronchodilators (SABA) following NICE guidelines [3]. GOLD guidelines suggest initial treatment of ICS should be reserved for patients in GOLD group D alongside LABA if blood eosinophil levels are greater than 300cells/μl. In addition, combination ICS (ICS/LABA) should be considered in patients who exacerbate if blood eosinophil levels are greater than 300 or 100 if they experience at least 2 moderate exacerbations or a hospitalization from AECOPD or remain breathless [4]. However, the use of ICS for the treatment of COPD has been debated.

FEV_1_ is a common measure used to assess lung function and multiple studies show FEV_1_ declines at a faster rate in smokers compared to non-smokers [5]. Randomised control trials (RCTs) have found that inhaled corticosteroids (ICS) reduce the rate of FEV_1_ decline in people with COPD [6–9]. However, the rate of FEV_1_ decline is heterogeneous and can vary depending on factors such as smoking status, exacerbations of COPD (AECOPD), and season [10, 11]. Most RCTs compare COPD patients on a specific ICS to those on a placebo, have specific inclusion and exclusion criteria and commonly exclude participants based on age, comorbidities and severity of disease [12]. In addition, most studies have short follow-up periods of less than 1 year. Therefore, most RCTs are not easily generalisable to the wider COPD population over the longer term.

Previous literature reviews have consisted of pre-specified ICS and non-ICS comparators [13–18] such as LAMA/LABA vs LABA/ICS or ICS, LAMA/LABA vs LABA, LAMA or LABA/ICS and more specific comparisons such as budesonide or beclomethasone vs placebo. Several large scale RCTs investigating various ICS and FEV_1_ decline have taken place since, such as the Study to Understand Mortality and Morbidity in COPD (SUMMIT) and the Withdrawal of Inhaled Steroids during Optimized Bronchodilator Management (WISDOM) trials, justifying the need to inform and summarise novel findings. We aimed to investigate the association between ICS or ICS-containing medications and FEV_1_ decline compared to non-ICS-containing medications in COPD and determine whether length of follow-up influences the difference in FEV_1_ decline between ICS and non-ICS containing groups.

## Material and methods

The systematic review protocol was registered with the International Prospective Register of Systematic Reviews (PROSPERO), registration number: CRD42018090741. For further detail on study protocol see study protocol in Additional file 1.

### Literature search

We systematically searched MEDLINE and EMBASE (up until the 25th April 2019) using the following key words:

1. COPD (Chronic Obstructive Pulmonary Disease); COAD (Chronic Obstructive Airways Disease); obstructive airflow/airway/lung/pulmonary/respiratory/bronchiectasis; emphysema; chronic bronchitis.
2. Inhaled corticosteroids; inhaled budesonide/fluticasone/beclomethasone/momentasone/flunisolide/ciclesonide.
3. Forced expiratory volume; lung function; respiratory function tests; FEV_1_; change/rate/decline/worse/reduce/decrease/slow FEV_1_/lung function/lung volume.

Searched terms included medical subject headings and free text words (see study protocol in Additional file 1). The Boolean operator “or” was used to search terms within the three concepts above and the operator “and” was used to combine the three concepts. Only English language literature was searched.

### Selection of studies

We included studies that had recruited people with physician diagnosed COPD or an FEV_1_/FVC < 70% who were aged 35 or older and were current or ex-smokers. Articles were included if the exposure and comparison were ICS-containing medications and non-ICS-containing medications and if they reported a change in FEV_1_ over time for both exposure and comparison groups. Change from baseline FEV_1_ was defined as change in post-bronchodilator FEV_1_. Articles were excluded if they included people with diagnosed asthma or asthma-COPD overlap syndrome.

Two reviewers (HW and MS) independently screened all titles and abstracts following the inclusion criteria and compared initial included titles. Any inconsistencies were discussed and if necessary, a third party intervened. This was repeated for full text articles. Conference papers, non-English language papers, review articles, protocols, or systematic reviews were not included.

### Data extraction, quality assessment and data synthesis

Data was extracted following predetermined criteria base on the PICO checklist. Study details included: study name; patient number; length of follow-up; study inclusion and exclusion criteria; population characteristics including recruitment method, gender, and mean age; non-ICS comparison; ICS type and dosage; crude and adjusted outcome (change in FEV_1_); statistical analysis; and any additional notes. Two reviewers extracted relevant data, which were compared and inconsistencies discussed.

Quality of studies was assessed using the Cochrane Risk of Bias Tool. This was developed to assess a RCTs external and internal validity [19]. This tool assesses selection bias, reporting bias, performance bias, detection bias, attrition bias, and biases not identified in the previous categories. Quality of studies were reported as high, moderate, or low bias.

A meta-analysis was performed to investigate treatment differences in change in FEV_1_ between ICS and non-ICS containing medications (Additional file 1: Figure S1). Treatment differences were calculated using t-tests [20]. Due to high heterogeneity (I^2^ = 98.9%; *P* < 0.0001) a descriptive synthesis was performed. Data were described with regards to type of ICS-containing medications, type of comparator, length of study follow-up and population characteristics.

## Results

**Fig. 1:**
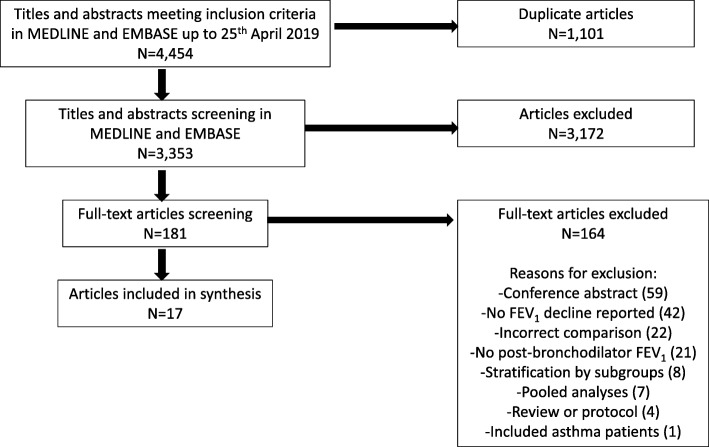
PRISMA flowchart illustrating selection process of articles

Four thousand four hundred fifty-four studies were identified in MEDLINE (*n* = 1319) and EMBASE (*n* = 3135) following the electronic systematic search. After duplicate articles were excluded 3353 article titles and abstracts were screened of which, 181 articles were selected for full text screening. Seventeen articles met our inclusion criteria illustrated in the PRISMA flowchart (Fig. 1). One hundred sixty-four articles were excluded (see Fig. 1 for further details).

### Study characteristics

All studies that met our inclusion criteria were RCTs (Table 1). Examples of RCTs that met our inclusion criteria included: ISOLDE (Inhaled Steroids in Obstructive Lung Disease in Europe), TRINITY, SUMMIT and TRISTAN (Trial of Inhaled Steroids and long-acting β_2_ Agonists).

**Table 1:** Study characteristics

Change in FEV_1_ Definition	Authors	Study Name	Geographic location	Follow-up (mths)	Patient N	Female (%)	Mean Age Years (SD)	Intervention (dose μg)	Change in FEV_1_ in ml (SD or 95%CI)
Mean change in FEV_1_ (ml)	Auffarth et al 1991 [21]	–	Netherlands	3	24	0.04	57.0 (8.2)	Placebo	-120 (230)
								Bud (1600)	15 (110)
	Cazzola et al 2000 [22]	–	Italy	3	80	11.6	64.2 (6.3)	Sal (50)	163 (80 to 245)
								Sal/FP (50/250)	188 (89 to 287)
								Sal/FP (50/500)	239 (183 to 296)
	Lee et al 2016 [23]	–	China, Hong Kong, Indonesia, South Korea, Thailand	3	577	4.3	66.8 (8.3)	Tio (18)	80 (27)
								Tio + bud/form (18 + 160/4.5)	160 (29)
	Bourbeau et al 1998 [24]	–	Canada	6	79	21.5	66.0 (8.0)	Placebo	0–3 months: −1(−65 to 62) 0–6 months:12(− 61 to 85)
								Bud (400)	0–3 months: −13(−59 to 33) 0–6 months:8(−51 to 68)
	Ohar et al 2014 [25]	–	United States, Argentina, Norway	6.5	639	46	62.9 (9.2)	Sal (50)	40 (342)
								FP/Sal (250/50)	140 (372)
	Vestbo et al 2005 [26]	TRISTAN	Australia, Austria, Belgium, Canada, Czech Republic, Denmark, Estonia, Finland, France, Germany, Greece, Hungary, Iceland, Italy, Lithuania, Netherlands, New Zealand, Norway, Poland, Russia, South Africa, Spain, Sweden, Switzerland, IK	12	1465	27.6	63.2 (8.6)	Placebo	−65 (−200 to 85)
								Sal (50)	0 (− 130 to 140)
								FP (500)	0 (−160 to 160)
								Sal/FP (50/500)	80 (−50 to 250)
Rate of FEV_1_ change (ml/year)	Vestbo et al 2017 [27]	TRINITY	Argentina, Belarus, Bulgaria, Croatia, Germany, Hungary, Italy, Mexico, Poland, Romania, Russia, Slovakia, Turkey, UK, Ukraine	12	2691	23.6	63.2 (8.6)	Tio (18)	21 (3 to 39)
								Fixed: Beclo/FP/gly bro (100/6/12.5)	82 (65 to 100)
								Open: Becl/FP/Tio (100/6/18)	85 (31 to 110)
	Wise et al 2000 [28]	Lung Health Study	North America, Canada	12	1116	36.9	56.3 (6.8)	Placebo	−47 (70.8)
								Triamcinolone acetonide (600)	−44.2 (69.8)
	Weir et al 1999 [29]	–	UK	24	98	25.5	66.6 (7.0)	Placebo	−56.9 (15)
								Becl (750)	−20.6 (16)
	Renkema et al 1996 [30]	–	Netherlands	24	59	0	56.0 (8.6)	Placebo	−60 (− 570 to 140)
								Bud (800)	−30 (−180 to 870)
								Bud + oral prednisolone (800/5)	−40 (−340 to 60)
	Burge PS et al 2000 [31]	ISOLDE	UK	36	751	25.4	63.7 (7.1)	Placebo	−59 (30.8)
								FP (500)	−50 (28.7)
	Calverley PM et al 2003 [32]	ISOLDE	UK	36	751	25.3	63.7 (7.1)	Placebo	−46
								FP (500)	−51
	Pauwels et al 1999 [33]	–	Belgium, Denmark, Finland, Italy, Netherlands, Norway, Spain, Sweden, UK	36	1277	27.2	52.5 (7.6)	Placebo	0–6 months: −81 9–36 months: −69
								Bud (400)	0–6 months: 17 9–36 months: −57
	Vestbo 1999 [34]	CCHS	Denmark	36	290	39.7	59.1 (9)	Placebo	−49.1
								Bud (400)	−46.0
	Calverley et al 2018 [35] & Vestbo et al 2016 [6]	SUMMIT	US, Argentina, Australia, Austria, Belarus, Belgium, Bosnia & Herzegovina, Bulgaria, Canada, Chile, China, Columbia, Croatia, Czech Republic, France, Georgia, Germany, Greece, Hungary, India, Indonesia, Israel, Italy, Japan, Korea, Latvia, Malaysia, Macedonia, Mexico, Netherlands, Philippines, Poland, Romania, Russia, Serbia, Slovakia, South Africa, Spain, Taiwan, Thailand, Turkey Ukraine, UK, Vietnam	48	16,485	25.5	65.0 (8.0)	Placebo	−46 (160.3)
								Vil (25)	−47 (154.0)
								FF (100)	−38 (154.3)
								FF/Vil (100/25)	−38 (154.1)
	Shaker et al 2009 [36]	–	Denmark	48	254	42	63.6 (7.4)	Placebo	−56 (−72 to −40)
								Bud (400)	−54 (−69 to-40)

Notes: 2 included studies (Calverley 2018, Vestbo 2016) were analyses on the same population and reported the same change in FEV_1_ estimates

Abbreviations: *FP* Fluticasone proprionate, *FF* Fluticasone furoate, *Sal* Salmeterol, *Bud* Budesonide, *Becl* Beclomethasone, *TIO* Tiotropium, *Vil* Vilanterol, *Mom* mometasone, *Form* formaterol., *UMEC* umeclidinium, *Gly Br* glycopyyronium bromide, *Ol* olodaterol

Included studies were published between 1991 to 2018; spanning a 27 year period. The number of patients included in studies ranged from 24 participants [21] to 16,485 patients [6, 35]. The majority of studies had high numbers of recruited males. The percentage of females in studies ranged from 0% [30] to 46% [25] and the median percentage of females included was 25.5%. The mean age of included participants ranged from approximately 53 years [33] to 67 years [23]. The median length of follow-up ranged from 3 months [21–23] to 4 years [6, 35].

Studies differed by types of ICS and non-ICS medications. The most common comparison was placebo vs ICS. Other comparisons included LABA vs LABA/ICS, placebo vs LABA/ICS, LABA vs ICS, and LAMA vs LAMA/ICS. Tables 1 and 2 illustrate all types of ICS and non-ICS comparisons in more detail.

**Table 2:** ICS and non-ICS-containing medication comparisons

Type of ICS-containing medication	Type of non-ICS containing medication	Number of studies
**Placebo**	**ICS**	**13**
	Fluticasone propionate	3
	Budesonide	6
	Fluticasone furoate	2
	Beclomethasone	1
	Triamicinolone acetonide	1
**LABA**	**LABA/ICS**	**5**
Salmeterol	Salmeterol/fluticasone proprionate	3
Vilanterol	Vilanterol/fluticasone furoate	2
**Placebo**	**LABA/ICS**	**3**
	Vilanterol/fluticasone furoate	2
	Salmeterol/fluticasone proprionate	1
**LABA**	**ICS**	**3**
Vilanterol	Fluticasone furoate	2
Salmeterol	Fluticasone proprionate	1
**LAMA**	**LAMA/ICS**	**2**
Tiotropium	Glycopyyronium/beclomethasone/fluticasone proprionate	1
Tiotropium	Tiotropium/beclothmethasone/fluticasone proprionate	1
**LAMA**	**LAMA + LABA/ICS**	**1**
Tiotopium	Tiotropium + formaterol/budesonide	1

Notes: numbers do not add up to the total number of studies included in the systematic review due to multiple ICS or non-ICS containing medications used in some studies. Medications in bold indicate higher level subgroups of ICS and non-ICS containing medications

Abbreviations: *ICS* Inhaled corticosteroid, *LABA* Long acting beta-agonist, *LAMA* Long acting muscarinic antagonist

### Change in FEV_1_

Table 1 illustrates the change in FEV_1_, by study and ordered by length of study follow-up, showing a high degree of variation between studies. A large proportion of the variation was dependent on study follow-up time and type of comparison. Change in FEV_1_ in studies that had less than one year of follow-up varied between − 120 ml (standard deviation [SD] 230) to + 163 ml (95% confidence intervals [CI] 80 to 245) over 3 months in non-ICS containing medications and between -13 ml (CI − 59 to 33) to + 239 ml (CI 183 to 296) over 3 months in ICS-containing medication [21, 22, 24]. Change in FEV_1_ in studies that had more than one year of follow-up varied between -69 ml/year to 21 ml/year (CI 3 to 39) in non-ICS containing medications and between -57 ml/year to 85 ml/year (CI 31 to 110) in ICS-containing medications [27, 33].

### Study follow-up time

**Fig. 2:**
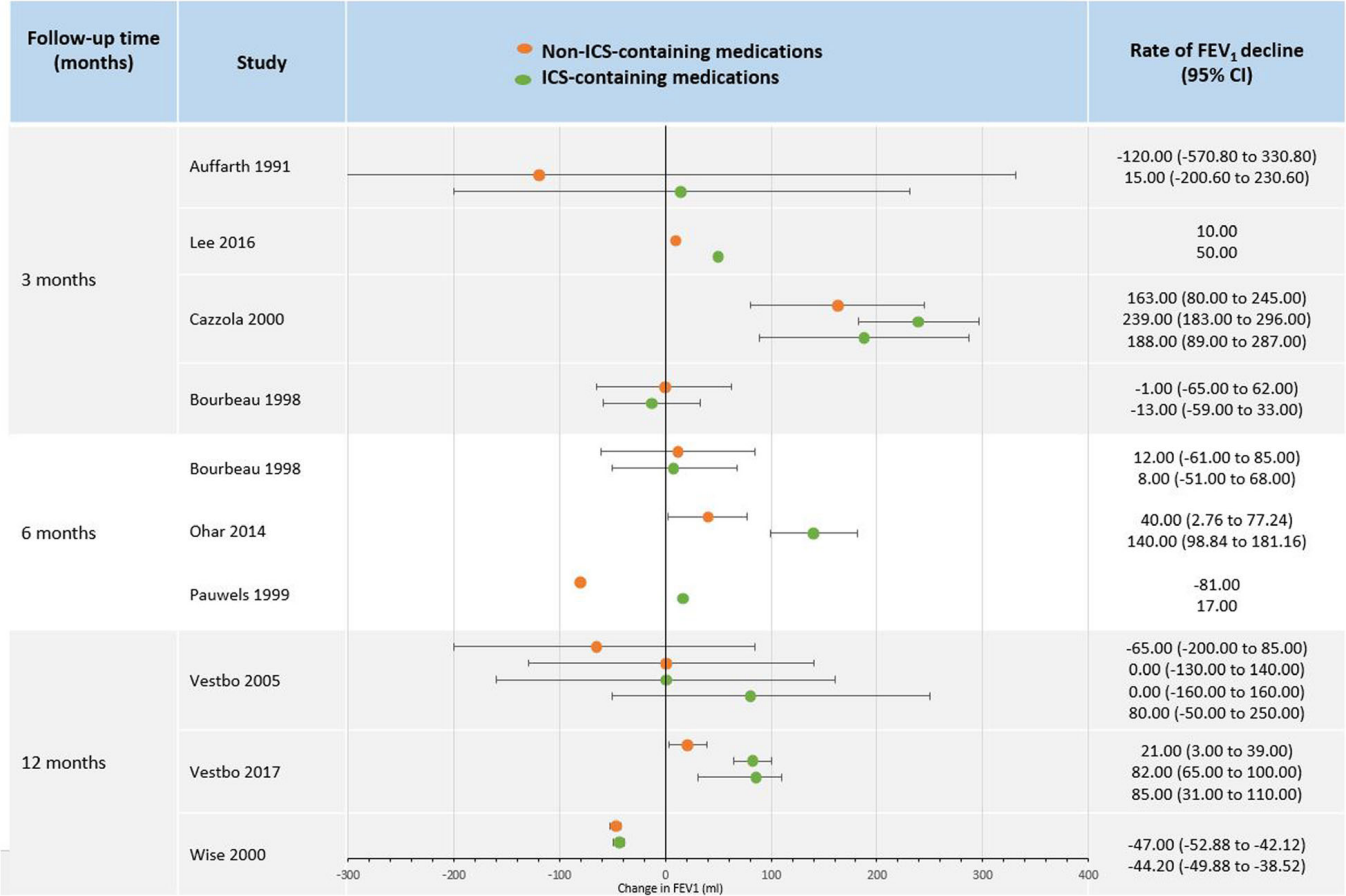
Change in FEV_1_ (ml) in studies with follow-up of one year or less. Studies are ordered by follow-up time. Note: Confidence intervals were not shown if the study did not report them or they were unable to be calculated

Figure 2 illustrates change in FEV_1_ in ICS and non-ICS-containing medications in studies with follow-up of one year or less. The majority of ICS point estimates show an increase in FEV_1_ and 8 out of 10 studies showed that change in FEV_1_ increased more or decreased slower in ICS groups compared to non-ICS groups.

**Fig. 3:**
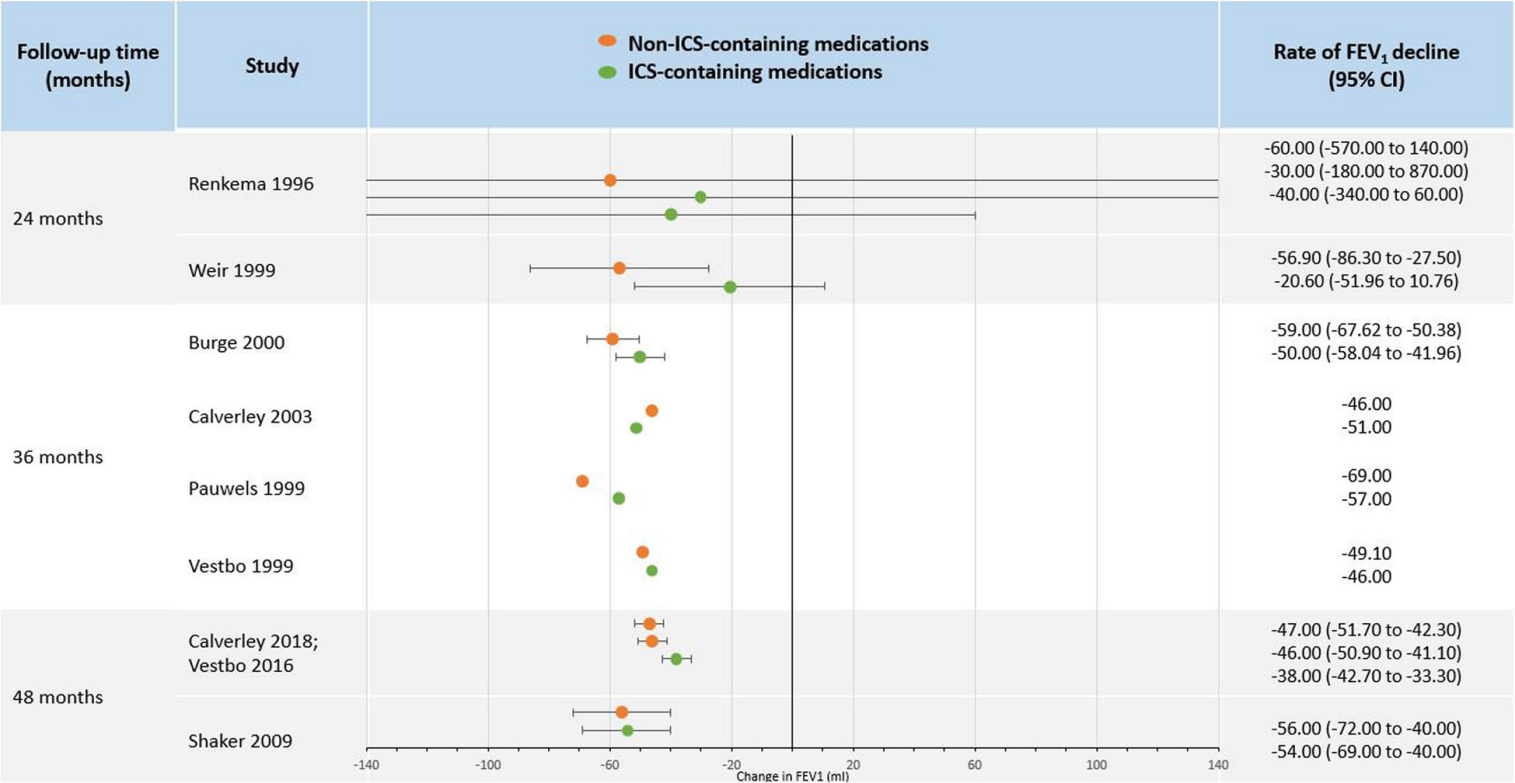
Change in FEV_1_ (ml/year) in studies with follow-up greater than one year. Studies are ordered by follow-up time. Note: Confidence intervals were not shown if the study did not report them or they were unable to be calculated

Figure 3 illustrates change in FEV_1_ in ICS and non-ICS-containing medications in studies with follow-up greater than one year in ml/year. All studies showed a decline in FEV_1_ in both ICS and non-ICS groups, of which there was little difference in FEV_1_ decline between the two groups.

The general trend in change in FEV_1_ with increasing follow-up time suggests that greater increases with ICS-containing medications are seen in short term studies up to approximately one year. Longer studies greater than a year show that FEV_1_ generally declines over time. All studies with greater than 1 year of follow-up were placebo vs ICS comparisons.

### Inclusion and exclusion criteria

Common inclusion criteria included specific criteria regarding age, smoking status and disease severity. Specifically, the majority of studies included patients aged 40 years old or older. In terms of smoking status, the majority of studies included current or ex-smokers with at least 10 pack years history smoking. Nearly all studies included patients with an FEV_1_/FVC < 70%. FEV_1_% predicted criteria was commonly 30–70% predicted or < 50%.

Furthermore, 4 studies required at least one AECOPD prior to the start of follow-up. These included moderate or severe AECOPD requiring prescribed oral corticosteroids and/or antibiotics or have been hospitalised for AECOPD prior to the start of the study. One study specifically required no AECOPD prior to study start. Other inclusion criteria included MRC dyspnea scores of 2 or more, FEV_1_ reversibility, and risk or history of cardiovascular disease. Additional file 1: Table S1 shows detailed inclusion criteria by study.

The most common exclusion criteria was the presence of diagnosed comorbidities including other respiratory diseases (e.g. asthma, pneumonia, URTI, LRTI) and clinically significant diseases that could affect results and patient participation (e.g. MI, HF, angina, and diabetes). Further exclusion criteria included long-term oxygen therapy, evidence of alcoholism or solvent abuse, AECOPD requiring prescription of oral corticosteroid, antibiotics, or hospitalisation prior to study start or moderate/severe AECOPDs. Additional exclusion criteria are shown in Additional file 1: Table S1.

### Quality assessment

The majority of studies were considered low risk in each of the bias domains. Reasons for considering ‘random sequence and allocation concealment’ unclear was due to no mention of a sequence generator in text or in additional files. ‘Reporting bias’ and ‘other biases’ were low risk because all outcomes mentioned in the methods were reported in the results. Similarly, no other biases were found in all studies. ‘Performance and detection bias’ was considered unclear in the study by Cazzola and colleagues [22] because the authors failed to report whether and how the study participants and personnel were blinded during follow-up and outcome assessment. ‘Performance and detection bias’ was considered high risk in the study by Lee and colleagues [23] as participants and personnel were not blinded during the study. The study by Shaker and colleagues [36] was considered to have unclear ‘attrition bias’ because there was no indication whether only participants with complete follow-up were used to measure change in FEV_1_. High risk ‘attrition bias’ was observed in 4 studies. This was because only participants with complete follow-up (i.e. completed the study and did not dropout) were included in the analysis. See Additional file 1: Figure S2 and Quality Assessment for quality assessment by risk of bias domain and support for judgment.

## Discussion

This systematic review investigated the change in FEV_1_ with ICS-containing medications compared to non-ICS-containing medications in COPD patients over the short and long term. Of the 17 studies that met our inclusion criteria, all were RCTs. We found that the majority of studies with less than a year follow-up reported increases in FEV_1_, with the general trend favouring ICS medications compared to non-ICS medications. Studies with more than a year follow-up generally reported a decline in FEV_1_ with little evidence of a treatment difference between ICS and non-ICS containing medications.

### Length of study follow-up

Our main finding suggests that initiating ICS medications improves lung function compared to non-ICS medications however, over long periods of time lung function declines at a similar rate in both ICS and non-ICS medications. This may be due to an initial acute bronchodilation, [37] or subtle improvements in care in both arms shortly after recruitment. The decline in FEV_1_ in studies greater than a year is seen in both ICS and non-ICS containing medications and raises the question of whether ICS-containing medications are similar to non-ICS medications over long periods of time with respect to their effect on lung function. In addition, the studies that reported a significant difference between the changes in FEV_1_ favouring ICS-containing medications were studies that were less than 1 year in duration.

### Type of ICS-containing medications and comparators

We found that in terms of rate of change of FEV_1_ per year, the majority of studies compared: i) placebos to monotherapy ICS; ii) LABA to LABA/ICS; iii) placebo to LABA/ICS; iv) LABA to monotherapy ICS; and v) LAMA to LAMA+LABA/ICS.

Previous literature suggests that ICS/LABA has better outcomes in COPD compared to the use of ICS monotherapy or LABA monotherapy. ICS/LABA is associated with reduced rate of AECOPD, improved FEV_1_ and improved patient health status compared to its individual components [38]. Barnes and colleagues showed that monotherapy ICS does not suppress inflammation in COPD [39, 40]. Further studies show that the anti-inflammatory effect of ICS is greater in the presence of beta agonists and help increase the number of beta-receptors and improve bronchodilation from LABA [41, 42]. Four studies in our systematic review included ICS/LABA as the ICS comparison arm. FEV_1_ improved in ICS/LABA groups compared to its non-ICS comparator whereas monotherapy ICS showed a decline in FEV_1_, similar to its non-ICS comparator. However, all studies investigating monotherapy ICS compared to a non-ICS medication had a follow-up greater than one year and all but one study investigating ICS/LABA had a follow-up of less than one year. All studies that compared ICS/LABA to LABA or ICS/LAMA to LAMA showed that FEV_1_ improved more in ICS combination groups compared to LABA or LAMA. Therefore, whilst improvement in FEV_1_ was seen in LABA and LAMA groups, the addition of ICS improved lung function further, highlighting the initial beneficial effect of ICS.

Furthermore, recently it has been suggested that the use of LAMA/LABA is preferential over ICS/LABA in COPD patients. This may be due to the synergistic effect of LABA and LAMA which activate both adrenergic and cholinergic pathways maximizing bronchodilation [43, 44]. A recent systematic review investigated the use of LAMA/LABA compared to ICS/LABA and found that compared to ICS/LABA, patients on LAMA/LABA had improved health status, decreased moderate or severe AECOPD, and decreased use of rescue medications [13, 16, 45]. Unfortunately, studies including LAMA/LABA were not included in the final synthesis as they did not meet our inclusion criteria so we were unable to investigate its effect on lung function compared to ICS/LABA.

Interestingly, the latest GOLD guidelines state that ICS/LABA use should be considered if blood eosinophils are greater than 300cells/μl in patients who exacerbate more frequently, severely, and who are more breathless [4]. Studies have shown that patients with high blood eosinophils who initiate ICS respond better in terms of lung function compared to those with low blood eosinophils [46]. On the other hand, observational studies have shown no association over longer follow-up periods [47]. As the studies we included did not stratify by eosinophils we were unable to look into this further however, further literature reviews and meta-analyses should explore this further.

### Strengths and limitations

This is an extensive literature update comparing the change in FEV_1_ between ICS-containing medications and non-ICS containing medications over time. ICS-containing medications were compared with non-ICS-containing medications in order to be as inclusive as possible and highlight differences in ICS type as well as length of follow-up and other study characteristics. The majority of studies included in this review had few biases and were of good quality. In addition, clinical trials with large patient populations such as TRISTAN, TRINITY, ISOLDE, and SUMMIT were included in this review.

One limitation of this systematic review is that ICS monotherapy was included even though it is not currently licensed in the UK [3, 48]. This is due to the risk of developing pneumonia and no improvement in lung function decline or risk of mortality compared to that of LABAs [49–51]. Over time prescribing ICS monotherapy has decreased [52] and it is advised by NICE that ICS monotherapy should not be used for treatment of COPD [48] . The majority of studies included reported a change in lung function in patients on ICS monotherapy, but 7 of the 12 studies were published in 2000 or earlier. The remaining studies that included ICS monotherapy were published between 2001 and 2018. These studies were either conducted in the United States or were multicenter studies that included centers in countries across Europe, Africa, and the Americas. Changes in FEV_1_ reported in these studies should therefore be interpreted with caution depending on the prescribing location.

Furthermore, whilst differences in change in FEV_1_ between ICS-containing medication and non-ICS-containing medications were seen, they were not always significant. This could have been due to small numbers of recruited patients. In addition, not all studies reported a treatment difference and it therefore unclear whether these differences are statistically significant as well as clinically significant. In those that did report statistical treatment differences, not all were clinically significant or vice versa. It has previously been suggested by the American Thoracic Society and the European Respiratory Society that a minimal important difference in FEV_1_ between two treatments ranges from 100 ml to 140 ml [53]. However, this is with regards to pharmacological trials and individual FEV_1_ measurements rather than a rate. In addition, it is important to note that clinically important differences in the real world may be different to those seen from RCTs.

Moreover, the results from included studies consist of mostly crude changes in FEV_1_. Whilst it is important to observe the range of crude changes with regards to ICS and non-ICS containing medications, they could be skewed by baseline FEV_1_. Milder patients with a higher baseline FEV_1_ may have more lung function to lose compared to a more severe patient with a lower baseline FEV_1_ [54]. Using a measure of change that accounts for baseline FEV_1_ may be more informative, such as percent change from baseline.

In addition, all studies included were RCTs and had many inclusion and exclusion criteria. All studies included patients with moderate to very severe COPD. Other common inclusion/exclusion criteria consisted of specific pack year smoking history and no other significant comorbidity. Whilst RCTs are important due to their valuable methodological design, they are typically not representative of the wider population of COPD patients, many of whom have comorbidities. Therefore, the representativeness of the results included in this review should be noted. Observational and general practice studies are needed to identify changes in lung function in a more representative COPD population with a wider degree of disease severity and comorbid conditions.

Lastly, we observed a high level of heterogeneity between studies and therefore, a network meta-analysis was not performed. This limited our ability to make conclusions on rate of change in FEV_1_ by ICS and non-ICS comparisons.

## Conclusion

The findings from this systematic review suggests that in COPD patients, initiating ICS medications improves lung function compared to non-ICS medications. However, over long periods of time lung function declines at a similar rate for both ICS and non-ICS medications. Further studies that are more generalizable to the wider population of COPD patients are needed in order to investigate the association between ICS and FEV_1_ decline further. Additionally, studies with a longer follow-up are needed to observe the long term effect of ICS on lung function.

## Additional file

**Additional file 1.** Systematic review protocol; **Figure S1:** Meta-analysis of treatment differences between ICS-containing medications and non-ICS-containing medications, stratified by follow-up time; **Table S1:** Inclusion and exclusion criteria of included studies; **Figure S2:** Quality assessment of included studies. Quality assessment.

## Data Availability

Not applicable.

## Abbreviations

COPD: Chronic obstructive pulmonary disease
FEV_1_: Forced expiratory volume in 1 s
ICS: Inhaled corticosteroids
LABA: long active beta agonist
LAMA: Long acting muscarinic antagonist
