# Supplementary material for: Inhaled corticosteroids and FEV1 decline in chronic obstructive pulmonary disease: a systematic review
Source: Respir Res. 2019 Dec 4;20:277. doi: 10.1186/s12931-019-1249-x (PMC6894275; doi:10.1186/s12931-019-1249-x)
Supplement: Supplementary file 1 — Additional file 1. Systematic review protocol; Figure S1: Meta-analysis of treatment differences between ICS-containing medications and non-ICS-containing medications, stratified by follow-up time; Table S1: Inclusion and exclusion criteria of included studies; Figure S2: Quality assessment of included studies. Quality assessment. [file 12931_2019_1249_MOESM1_ESM.docx]

Supplementary material

**Table of Contents**

[**Systematic review protocol** 1](#_Toc21609578)

[**Figure S1:** Meta-analysis of treatment differences between ICS-containing medications and non-ICS-containing medications, stratified by follow-up time 7](#_Toc21609579)

[**Table S1**: Inclusion and exclusion criteria of included studies 8](#_Toc21609580)

[**Figure S2**: Quality assessment of included studies 11](#_Toc21609581)

[**Quality assessment: Support for judgement** 12](#_Toc21609582)

# **Systematic review protocol**

**Title:** Inhaled corticosteroids and FEV_1_ decline in COPD: a protocol for a systematic review and meta-analysis

**Background**

***Rationale***Chronic obstructive pulmonary disease (COPD) is a progressive disease that is not fully reversible and is characterised by obstruction of airflow in the lungs (1). COPD was ranked as the ninth leading cause of death worldwide in 2016 and is projected to be the third leading cause of death globally by 2020 (2). FEV_1_ is a common measure used to asses lung function, and since the work by Fletcher and Peto it is understood that lung function declines at a faster rate in smokers compared to non-smokers (3).

Previous randomised control trials (RCTs) have found that inhaled corticosteroids (ICS) reduce the rate of FEV_1_ decline in people with COPD (4-7). However, the rate of FEV_1_ decline is heterogeneous and can vary depending on factors such as smoking status, AECOPD, and seasonality (8, 9). The majority of studies on ICS and rate of FEV_1_ decline have been randomised control trials (RCTs), of which most compare COPD patients on a specific ICS to those on a placebo. RCTs have specific inclusion and exclusion criteria and commonly exclude participants based on age, comorbidities and severity of disease (10). In addition, most studies have short follow-up periods of approximately less than 1 year. Therefore RCTs limit the generalisability of findings to the wider COPD population. Whilst they are an important study design in research, it is essential that high quality observational studies are performed alongside RCTs. To date, no systematic reviews investigating the relationship between ICS-containing medication and rate of FEV_1_ decline in COPD have included both RCTs and observational studies and compared their findings.

***Objective****s*We aim to investigate the association between ICS or ICS-containing medication and FEV_1_ decline in COPD patients in RCTs and observational studies independently and compare overall findings.

**Methods**
Preferred Reporting Items for Systematic Reviews and Meta-Analyses Protocols (PRIMSA-P) 2015 guidelines were used in developing this systematic review protocol(11).

***Eligibility Criteria***

*Participants:* People with a physician diagnosis of COPD or who have an FEV_1_/FVC<70%, who are 35 years old or older and are smokers or ex-smokers will in included.

*Study designs:* Prospective observational studies and RCTs will be included. Patients must have at least 2 FEV_1_ measurements during study follow-up.

*Exposure*: The exposure for this systematic review is ICS-containing medication. ICS-containing medication can include ICS monotherapy, or combination ICS therapy. Combined ICS therapy can include ICS combined with a long-acting beta agonist, a long acting muscarinic antagonist, a short acting beta agonist, and a short acting muscarinic antagonist.

*Comparisons:* Studies should compare people with COPD on an ICS-containing medication with people on a placebo or non-ICS-containing medication. For randomised control trials we will include studies that compare people with COPD on the intervention (mono or combined ICS) to people with COPD on a placebo, or any other non-ICS COPD medications. In observational studies we will include studies that compare people with COPD on an ICS-containing medication to people with COPD not on any ICS-containing medication.

*Outcomes:* Our outcome of interest is rate of FEV_1_ decline. Studies will be considered if the outcome is expressed as change in FEV_1_ over time. Units can include millilitres or litres per year, change in percentage baseline FEV_1_ (%), and absolute change in FEV_1_ in millilitres (ml) from baseline.

*Exclusion criteria:* Conference presentations available as an abstract but not as a full paper will be excluded. In addition, only English language papers will be included.

***Information sources***
Journal databases will be used to search for relevant articles. MEDLINE and EMBASE will be searched using the journal database platform, OVID. In order to keep up to date with the current systematic review literature, the Cochrane Database of Systematic Reviews and PROSPERO will be regularly searched for systematic reviews or protocols on ICS-containing medication and FEV_1_ decline in people with COPD. Literature will be searched up until the 30^th^ November 2018.

***Search strategy***
Medical subject headings and text words will be used to identify literature related to COPD, ICS-containing medication, and rate of FEV_1_ decline. These three concepts will be combined using the Boolean operator “AND” to search for potentially relevant literature. An example of the medical subject headings and text words used in our search are shown in table 1.

Table 1: Example of literature search in MEDLINE

| Concept 1: COPD | Pulmonary disease, chronic obstructive/ |
| --- | --- |
|  | COPD.mp |
|  | COAD.mp |
|  | Obstruct$ adj3 (airflow$ or airway$ or lung$ or pulmonary or respiratory or bronch$).mp |
|  | Emphysema$.mp |
|  | Chronic$ adj3 bronch$.mp |
| Concept 2: ICS | Inhal$ corticosteroid$.mp |
|  | Inhal$ adj3 corticosteroid$.mp |
|  | Ics.mp |
|  | Inhal$ aj3 (budesonide or fluticasone or beclomethasone or mometasone or flunisolide or ciclesonide).mp |
| Concept 3: Lung function decline | Forced expiratory volume/ |
|  | Lung function/ |
|  | Respiratory function tests/ |
|  | FEV1.mp |
|  | (Chang$ or rate$ or declin$ or worse$ or reduc$ or decreas$ or slow$) adj3 (FEV1 or lung$ function or lung$ volume$) |

***Study records****:
Data management:* Selected literature will be organised using Endnote and an excel spreadsheet including titles and relevant information extracted from the article.

*Selection process:* The selection of relevant literature will be performed in two steps. Firstly the primary reviewer will review all titles and abstracts using the inclusion criteria stated above. The second reviewer will review a proportion of titles and abstracts. The reviewers will compare included titles and discuss any inconstancies. If necessary a third person will discuss discrepancies. Uncertain titles will be included to minimise the risk of rejecting a relevant article.

Thereafter, full texts of the included titles will be reviewed by the reviewers and checked against the inclusion criteria. Both reviewers will review all full texts. If information in the literature is not clear supplementary material will be used and authors may be contacted. Those full texts meeting the inclusion criteria will be included in the systematic review. A list of rejected articles will be recorded.

*Data collection process:* Data and information from included articles will be recorded into an excel spreadsheet. Lists of article titles and extracted information will be compared between reviewers and duplicated articles will be identified.

***Data items***
Data will be extracted from included literature using the PICO checklist. Information on study populations, interventions, control groups, and outcomes will be extracted. Specifically, study design, length of study, population, COPD diagnosis criteria, ICS type, number of FEV_1_ measurements, outcome definition, details on statistical analysis, and the outcome data. Both crude and adjusted outcome measures will be recorded as well as covariates used. If outcome measures are not included in the full text or supplementary material authors will be contacted in order to obtain these measures. Data will be extracted using an excel extraction tool where information listed above will be stored. This data extraction tool will be piloted before it is used on all included literature.

***Outcomes and prioritisation***
The outcome of interest for this systematic review is rate of lung function decline, specifically rate of FEV_1_ decline. Rate of FEV_1_ decline measured in ml or L per year or month or any specific time period will be prioritised. Other outcome measures considered will include change in percentage baseline FEV_1_ (%), and absolute change in FEV_1_ (ml/L) from baseline if baseline FEV_1_ is reported.

***Risk of bias in individual studies***
Risk of bias assessment will be performed separately for randomised control trials and observational studies. For randomised control trials we will use the Cochrane Risk of Bias Tool. This assesses selection bias, reporting bias, performance bias, detection bias, and attrition bias.

To assess the risk of bias in observational studies we will use the Risk of Bias in Non-randomised Studies-of Interventions (ROBINS-I) tool. This tool is suitable because we are interested in comparing rate of FEV_1_ decline in those on an ICS-containing medication to those not on an ICS-containing medication in the observational that do not randomise participants. ROBINS-I assesses pre-intervention biases, such as confounding and selection bias, biases at intervention such as classification of interventions, and post-intervention biases, such as missing data, outcome measurements and reporting bias.

All domains will be identified as high, moderate, or low bias and an overall risk of bias assessment will be made and reported in the review. Two reviewers will independently assess the risk of bias in all included studies.

***Data synthesis***

The aim of this systematic review is to compare the FEV_1_ decline in people with COPD on an ICS-containing medication with people with COPD either on a placebo, or non-ICS-containing medication. A descriptive synthesis will be provided for RCTs and observational studies separately including type of ICS-containing medication, outcome definitions, and population characteristics. We additionally aim to produce a network meta-analysis for both RCTs and observational studies separately using random-effects network meta-analysis and treatment differences in rates of FEV_1_ decline. Studies with heterogeneity greater than 50% will be considered as moderately to highly heterogeneous(12). Furthermore, we aim to stratify by severity of COPD, quality of study, and length of study to investigate heterogeneity further.

***Risk of bias in meta-anaysis***
Where possible we will create funnel plots by plotting effect estimates against standard errors and Begg’s test for asymmetry will be used in order to determine reporting bias in the meta-analyses. If the number of included studies are small we will discuss biases across the studies and highlight this as a limitation in our conclusions.

***Confidence in cumulative evidence***The quality of evidence of included literature will be assessed using the GRADE guidelines (13). This technique grades studies based on our confidence in the effect measurement. Studies can be graded as high (the true effect is close to the effect estimate), moderate (reasonably confident that the true effect is close to the effect estimate), low (the true effect may be considerably different to the effect estimate), very low (highly likely that the true effect is considerably different to the effect estimate). RCTs are considered as high quality at the first instance and observational studies are considered as low quality. Factors including risk of bias assessment, inconsistency, indirectness, imprecision, publication bias, and effect magnitude will contribute to the final grading of the quality of evidence. Quality of evidence will be reported.

**Ethics and dissemination**Due to the nature of the study ethical approval is not needed. We aim to publish our findings in a peer-reviewed journal and present our work at conferences both nationally and internationally.

***Limitations***The main limitation of this study will be heterogeneity between studies. Our broad search strategy retrieves any combination of ICS therapy as well as RCTs and observational studies. If the data allows we aim to perform a network meta-analysis and descriptive analyses will be stratified by study characteristics.

***Conclusion***We aim to improve the understanding of the association between ICS-containing medications and rate of FEV_1_ decline in COPD with an updated review of the existing literature.

# **Figure S1:** Meta-analysis of treatment differences between ICS-containing medications and non-ICS-containing medications. Treatment differences are means (95% CI).


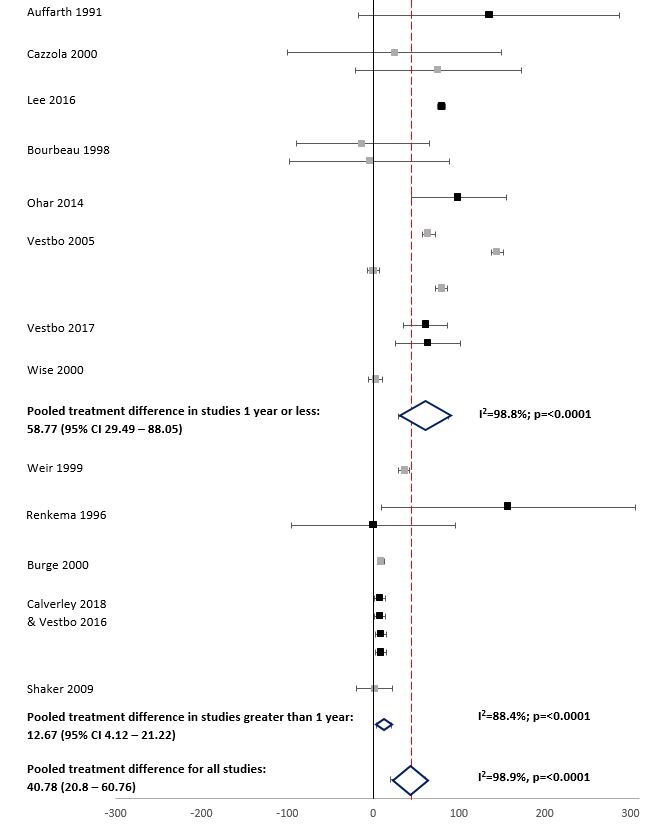


Treatment difference (ml)

Notes: 3 studies did not report standard deviations or 95% CI for treatments and were excluded from the meta-analysis.

# **Table S1**: Inclusion and exclusion criteria of included studies

| Author | Inclusion criteria | Exclusion criteria |
| --- | --- | --- |
| Calverley PM 2003 | Age 40-74; clinical diagnosis of COPD; history of current/previous smoking; FEV_1_ <85% predicted; FEV_1_/FVC <70%; FEV_1_ ≥0.8L | Clinical diagnosis asthma; require non-trial anti-inflammatory treatment for lung disease or β-adrenoblockers; <5 years life expectancy due to concomitant disease; unable to meet required standards for spirometry |
| Auffarth 1991 | Smoking ≥1 cigarette/day for at least 5 years; FEV1 30-75% predicted; reversibility less than 20% FEV1 predicted; provocative concentration of histamine causing a 20% fall in FEV_1_ less than 16mg/ml; negative skin test response to 12 allergens/IgE for house dust mite; total serum IgE below 470IU/ml; blood eosinophils below 0.2x10^6^/L; no URTI or ocs 2 months prior to start; ICS stopped 2 weeks prior to screening |  |
| Bourbeau 1998 | Age ≥40; smokers or ex-smokers; no asthma history in childhood or adulthood; no AECOPD in last 2 months; pre-bronchodilator FEV1<65% predicted; FEV1/FVC<0.65; post-bronchodilator FEV1 >80% predicted; regular treatment with ≥1 bronchodilator; no ICS or ocs in previous 2 months; no other active lung disease or diabetes, peptic ulcer disease, uncontrolled high blood pressure; hf, any disease other than COPD that would affect qol; non-responders to ocs |  |
| Vestbo 2017 | Age ≥40; current or ex-smoker; COPD diagnosis with post bronchodilator FEV1<50% predicted; FEV1/FVC<0.7; ≥1 moderate/severe AECOPD in prior year; used ICS +LABA or ICS+LAMA or LAMA monotherapy for ≥2 months prior to screening | ICS/LAMA/LABA 2 months prior to screening; asthma diagnosis; allergic rhinitis or atrophy; AECOPD 4 weeks prior; clinically significant cardiovascular conditions or laboratory abnormalities; unstable concurrent disease that could influence safety and efficacy |
| Burge 2000 | Age 40-75; current or former smokers; non-asthma COPD; FEV1 post bronchodilator >0.8L and <85% predicted; FEV1/FVC<70% | FEV1 response to 400ug salbutamol exceeding 10% predicted; life expectancy <5 years; concurrent diseases; used β-blockers |
| Pauwels 1999 | Age 30-65; current smokers smoking at least 5 cigarettes/day, smoked cigarettes for >10 years, smoking history ≥5 pack years; FEV1 50-100% predicted; FEV1/FVC<70%; increase in FEV1 after inhalation of 1mg terbutaline <10%; change in FEV1 between the end of first 3 months of run in and end of second <15% | History of asthma, allergic rhinitis, allergic eczema; oral glucocorticoids for >4 weeks during 6 months follow-up |
| Vestbo 1999 | Age 30-70; FEV1/FVC<0.7; FEV1 reversibility after inhalation of 1mg terbutaline<15%; FEV1 reversibility after 10 days treatment with oral prednisolone<15%; informed consent | Long term treatment (>2 episodes of >4 weeks) ocs or ICS within 6 months of study entry; pregnancy or lactation; intention to become pregnant; other serious systemic disease that could influence results; chronic alcohol and drug use; participation in other clinical studies of COPD within 1 month of inclusion |
| Ohar 2014 | Age ≥40; AECOPD within 2 weeks of start and hospitalised for ≤1 days or emergency room observation for ≥24hours with ocs & abx treatment or visit to GP or emergency room for <24 hours with ocs & abx treatment plus 6 months history of AECOPD related hospitalisations | Other significant co-morbid conditions (current or history) including asthma, lung cancer, uncontrolled diabetes and hypertension, angina etc., abnormal ECG or chest x-rays at visit 1, pregnancy, physical disability, hypersensitivity to β_-_agonists, any adverse reaction, substance abuse or psychiatric disease that might interfere with study |
| Calverley 2018  & Vestbo 2016 | Age 40-80; current or former smokers with ≥10 pack-years; post bronchodilator FEV1 50%-70% predicted; FEV1/FVC<0.7; MRC≥2; history or at risk of CVD | Current diagnosis of asthma; significant lung disease other than COPD; lung reduction surgery; receiving long term O_2_ therapy or ocs; severe hf; life expectancy less than 2 years; end-stage chronic renal disease |
| Cazzola 2000 | Age ≥50; well controlled COPD; 20≤ smoking pack years; change in FEV1≤12% predicted following salbutamol 400ug; post-bronchodilator FEV1<85%; good MDI technique; previously been individually dose titrated with SR theophylline to serum theophylline level 10-20 ug/mL | Asthma as primary diagnosis; unstable respiratory disease requiring oral/parenteral steroids within 4 weeks prior to start; upper or lower RTI within 4 weeks of screening visit; unstable angina or unstable arrythmia; concurrent use of medications that affected COPD or interact with methylxanthine products (macrolides or fluoroquinolones, evidence of alcohol abuse) |
| Vestbo 2005 | Age 40-79; COPD by ERS definition; >10 smoking pack years; pre-bronchodilator FEV1 25-70% predicted; FEV1/FVC<70%; poor short term reversibility (<10% predicted FEV1 30 mins post 400ug salbutamol); chronic bronchitis with exacerbations in the last three years | Current diagnosis asthma; eczema; allergic rhinitis; treatment with systemic steroids, antibiotics; change in COPD medication in last 4 weeks; use of SABAs, other ICSs, other LABAs and combination bronchodilators (Combivent, berodual, duovent) |
| Renkema 1996 | Clinical diagnosis of COPD based on history (persistent dyspnoea, on exertion, without sudden attacks of dyspnoea); FEV1<80% predicted ;RV>100% predicted; specific compliance (Csp%pred)>100% post BD - if air trapping> 1.5L Csp allowed to be <100%pred; no signs of allergy (negative SPT, <200IU/mL IgE, <250x10^3^/mL peripheral blood eosinophils; serum α_1_-anti-trypsin level within normal range; clinically stable disease | >70 years old at entry; receiving continuous corticosteroid therapy; severe concomitant disease which may interfere with the study |
| Lee 2015 | Age ≥40 years; clinical diagnosis of COPD with symptoms for >2 years; history of ≥1 AECOPD requiring steroids/abx within 1-12 months; current or prior smoking history of ≥10 pack years; pre-bronchodilator FEV1 ≤50% predicted; pre-bronchodilator FEV1/FVC <70% | History of asthma; history of seasonal allergic rhinitis before age 40; AECOPD requiring hospitalisation/A&E admission 4 weeks prior to or during run-in period; used systemic/inhaled glucocorticosteroids 4 weeks/2 weeks before run-in period; significant cardiovascular disorder; significant respiratory tract disorder (not COPD); received non-cardio selective oral or ophthalmic β-blocking agents; narrow-angle glaucoma; prostatic hyperplasia; bladder neck obstruction |
| Shaker 2009 | Aged 50-80; current smokers with history of ≥10 cigarettes a day during last 6 months and previous history of ≥20 pack years; clinical diagnosis of COPD for <2 years; FEV1 35-70% predicted; FEV1/FVC≤60% | Ex-smokers; FEV1 reversibility ≥12% and 200ml from baseline 15 mins after 1mg terbutaline inhalation or ≥15% and 300ml after 2 weeks on oral prednisolone (25mg); any severe concomitant disease; AECOPD 30 days prior to first visit; ocs for >4 weeks 6 months prior to first visit; long-term O_2_ therapy |
| Wise 2000 | Age 40-69; FEV1/FVC<0.7; FEV1 30-90% predicted; current smokers or ex-smokers who had quit within previous 2 years | Any other medical conditions; recent mi, alcoholism; hf; insulin-dependent diabetes mellitus; neuropsychiatric disorders; bronchodilator use or ics/ocs in previous year |
| Weir 1999 | Clinical diagnosis COPD; adult onset airflow obstruction; FEV1<70% predicted; FEV1/FVC<65% | Diagnosis of asthma; clinically significant bronchodilator reversibility; history of acute attacks of breathlessness and recovery between episodes; significant improvement with steroid use in the past; patients who thought that steroid treatment was clinically indicated; prescribed ocs ≥3 months in previous year or anytime in 4 weeks before trial |

# **Figure S2**: Quality assessment of included studies

| Study Authors | Random sequence | Allocation concealment | Reporting bias | Other bias | Performance bias | Detection bias | Attrition bias |
| --- | --- | --- | --- | --- | --- | --- | --- |
| Calverley 2003 | Unclear | Unclear | Low | Low | Low | Low | Low |
| Auffarth 1991 | Unclear | Unclear | Low | Low | Low | Low | High |
| Bourbeau 1998 | Low | Low | Low | Low | Low | Low | Low |
| Vestbo 2017 | Low | Low | Low | Low | Low | Low | Low |
| Burge 2000 | Low | Low | Low | Low | Low | Low | Low |
| Pauwels 1999 | Unclear | Unclear | Low | Low | Low | Low | Low |
| Vestbo 1999 | Low | Low | Low | Low | Low | Low | Low |
| Ohar 2014 | Low | Low | Low | Low | Low | Low | Low |
| Calverley 2018 & Vestbo 2016 | Low | Low | Low | Low | Low | Low | Low |
| Cazzola 2000 | Low | Unclear | Low | Low | Unclear | Unclear | Low |
| Vestbo 2005 | Low | Low | Low | Low | Low | Low | High |
| Renkema 1996 | Low | Low | Low | Low | Low | Low | Low |
| Lee 2015 | Unclear | Unclear | Low | Low | High | High | Low |
| Shaker 2009 | Low | Low | Low | Low | Low | Low | Unclear |
| Wise 2000 | Low | Unclear | Low | Low | Low | Low | High |
| Weir 1999 | Unclear | Unclear | Low | Low | Low | Low | High |

# **Quality assessment: Support for judgement**

| **Author & year:** Calverley 2003 | | |
| --- | --- | --- |
| **Domain** | **Risk of bias** | **Support for judgement** |
| **Random sequence generation**  *Selection bias* | Unclear | Quote: “Treatment was randomized”  Comment: Probably done however, more detail is needed |
| **Allocation concealment** *Selection bias* | Unclear | Did not mention |
| **Selective reporting** *Reporting bias* | Low | All outcomes measures listed in methods section were reported in results |
| **Other sources of bias** *Other bias* | Low | No other sources of bias |
| **Blinding of participants and personnel** *Performance bias* | Low | Quote: “Double blind”  Comment: Probably done |
| **Blinding of outcome assessment** *Detection bias* | Low | Quote: “Double blind”  Comment: Probably done |
| **Incomplete outcome data** *Attrition bias* | Low | While 156/376 patients withdrew from ICS and 193/375 withdrew from placebo, all patients were included in analyses and patients who withdrew were compared with patients who completed the study in terms of outcomes |

| **Author & year:** Auffarth 1991 | | |
| --- | --- | --- |
| **Domain** | **Risk of bias** | **Support for judgement** |
| **Random sequence generation**  *Selection bias* | Unclear | Quote: “They were then allocated at random to one of two parallel groups in a double blind design”  Comment: Probably done but no description of how it was done |
| **Allocation concealment** *Selection bias* | Unclear | Not mentioned |
| **Selective reporting** *Reporting bias* | Low | All outcomes measures listed in methods section were reported in results |
| **Other sources of bias** *Other bias* | Low | No other sources of bias |
| **Blinding of participants and personnel** *Performance bias* | Low | Quote: “double blind design”  Comment: Probably done |
| **Blinding of outcome assessment** *Detection bias* | Low | Quote: “double blind design”  Comment: Probably done |
| **Incomplete outcome data** *Attrition bias* | High | Quote: “Eleven of the 12 placebo treated and 10 of the 12 budesonide treated patients completed the trial”.  Comment: ITT patients used for baseline characteristics, FEV% predicted however, complete population used for PC20 ratio, and n=12 (ICS) and n=11 (placebo) for diary card data. |

| **Author & year:** Bourbeau 1998 | | |
| --- | --- | --- |
| **Domain** | **Risk of bias** | **Support for judgement** |
| **Random sequence generation**  *Selection bias* | Low | Quote: “Patients were randomly assigned”; “Randomisation was carried out in blocks of four patients to ensure similar numbers of patients in each treatment group”  Comment: Probably done |
| **Allocation concealment** *Selection bias* | Low | Quote: “Identification of individual treatment assignments was only possible in case of emergency by breaking the sealed envelope kept by the investigator. The envelopes had to be kept with the case record forms and be returned unbroken at the end of the study”  Comment: Probably done |
| **Selective reporting** *Reporting bias* | Low | All outcomes measures listed in methods section were reported in results |
| **Other sources of bias** *Other bias* | Low | No other biases |
| **Blinding of participants and personnel** *Performance bias* | Low | Quote: “double blind”  Comment: Probably done |
| **Blinding of outcome assessment** *Detection bias* | Low | Quote: “double blind”  Comment: Probably done |
| **Incomplete outcome data** *Attrition bias* | Low | Quote: “All the analyses were performed on an intention-to-treat basis, meaning that all patients randomised to treatment were included in the analysis, regardless of protocol violations, and including those who had to be withdrawn up to the point of withdrawal”  3/39 withdrew from ICS, 10/40 withdrew from placebo  Comment: Probably done |

| **Author & year:** Vestbo 2017 | | |
| --- | --- | --- |
| **Domain** | **Risk of bias** | **Support for judgement** |
| **Random sequence generation**  *Selection bias* | Low | Quote: “Patients were randomised to treatment by investigators contacting an interactive response technology (IRT) system, which used a randomisation list generated by the IRT provider. Randomisation was in the ratio 2:2:1”  Comment: Probably done |
| **Allocation concealment** *Selection bias* | Low | Quote: “Patients, investigators, site staff, and funder personnel were masked to treatment assignment for the duration of  the study”  Comment: Probably done |
| **Selective reporting** *Reporting bias* | Low | All outcomes measures listed in methods section were reported in results |
| **Other sources of bias** *Other bias* | Low | No other sources of bias |
| **Blinding of participants and personnel** *Performance bias* | Low | Quote: “double-  blind, double-dummy  Quote: “double-  blind, double-dummy  double-  blind, double-dummy  double-  blind, double-dummy  Quote: “double blind, double dummy”  Comment: Probably done |
| **Blinding of outcome assessment** *Detection bias* | Low | Quote: “double blind, double dummy”  Comment: Probably done |
| **Incomplete outcome data** *Attrition bias* | Low | Quote: “We analysed primary, key secondary, and other secondary endpoints in the intention-to-treat population”  92/1077 withdrew from fixed triple, 161/1075 withdrew from non-ICS group, 42/538 withdrew from open triple  Comment: Probably done |

| **Author & year:** Burge 2000 | | |
| --- | --- | --- |
| **Domain** | **Risk of bias** | **Support for judgement** |
| **Random sequence generation**  *Selection bias* | Low | Quote: “We used a computer generated allocation schedule stratified by centre (block size of six). Patients were randomised sequentially from a list comprising treatment numbers only”  Comment: Probably done |
| **Allocation concealment** *Selection bias* | Low | Quote: “Patients were randomised sequentially from a list comprising treatment numbers only”  Comment: Probably done |
| **Selective reporting** *Reporting bias* | Low | All outcomes measures listed in methods section were reported in results |
| **Other sources of bias** *Other bias* | Low | No other sources of bias |
| **Blinding of participants and personnel** *Performance bias* | Low | Quote: “double blind”  Comment: Probably done |
| **Blinding of outcome assessment** *Detection bias* | Low | Quote: “double blind”  Comment: Probably done |
| **Incomplete outcome data** *Attrition bias* | Low | Quote: “Analyses for each parameter included all randomised patients with at least one valid measurement”  160/376 withdrew from ICS, 195/375 withdrew from placebo  Comment: Complete case analysis |

| **Author & year:** Pauwels 1999 | | |
| --- | --- | --- |
| **Domain** | **Risk of bias** | **Support for judgement** |
| **Random sequence generation**  *Selection bias* | Unclear | Not stated |
| **Allocation concealment** *Selection bias* | Unclear | Not stated |
| **Selective reporting** *Reporting bias* | Low | All outcomes measures listed in methods section were reported in results |
| **Other sources of bias** *Other bias* | Low | No other sources of bias |
| **Blinding of participants and personnel** *Performance bias* | Low | Quote: “double blind”  Comment: Probably done |
| **Blinding of outcome assessment** *Detection bias* | Low | Quote: “double blind”  Comment: Probably done |
| **Incomplete outcome data** *Attrition bias* | Low | Quote: “Data on the randomized subjects were analyzed on an intention-to-treat basis”  176 withdrew from ICS, 189 withdrew from placebo  Comment: complete case analysis |

| **Author & year:** Vestbo 1999 | | |
| --- | --- | --- |
| **Domain** | **Risk of bias** | **Support for judgement** |
| **Random sequence generation**  *Selection bias* | Low | Quote: “Randomisation was masked and the randomisation sequence generated by computer at Astra. Study numbers were allocated in a consecutive order”  Comment: Probably done |
| **Allocation concealment** *Selection bias* | Low | Quote: “The randomisation code was held by Astra and was not available to the researchers until the study had been completed”  Comment: Probably done |
| **Selective reporting** *Reporting bias* | Low | All outcomes measures listed in methods section were reported in results |
| **Other sources of bias** *Other bias* | Low | No other sources of bias |
| **Blinding of participants and personnel** *Performance bias* | Low | Quote: “double blind”  Comment: Probably done |
| **Blinding of outcome assessment** *Detection bias* | Low | Quote: “double blind”  Comment: Probably done |
| **Incomplete outcome data** *Attrition bias* | Low | Quote: “only intention-to-treat results are shown”  Comment: Both ITT and per protocol population were used but only ITT population were shown. |

| **Author & year:** Ohar 2014 | | |
| --- | --- | --- |
| **Domain** | **Risk of bias** | **Support for judgement** |
| **Random sequence generation**  *Selection bias* | Low | Quote: “Allocation of double-blinded study treatments was conducted using RAMOS (GlaxoSmithKline, UK), an interactive voice response system”  Comment: Probably done |
| **Allocation concealment** *Selection bias* | Low | Quote: “Allocation of double-blinded study treatments was conducted using RAMOS (GlaxoSmithKline, UK), an interactive voice response system”  Comment: Probably done |
| **Selective reporting** *Reporting bias* | Low | All outcomes measures listed in methods section were reported in results |
| **Other sources of bias** *Other bias* | Low | No other biases |
| **Blinding of participants and personnel** *Performance bias* | Low | Quote: “double-blind”  Comment: probably done |
| **Blinding of outcome assessment** *Detection bias* | Low | Quote: “double-blind”  Comment: probably done |
| **Incomplete outcome data** *Attrition bias* | Low | Quote: “All efficacy and safety analyses were performed in the intent-to-treat (ITT) population”  26/314 withdrew from ICS; 39/325 withdrew from non-ICS  Comment: Randomised population used |

| **Author & year:** Calverley 2018 | | |
| --- | --- | --- |
| **Domain** | **Risk of bias** | **Support for judgement** |
| **Random sequence generation**  *Selection bias* | Low | Quote: “Participants were randomly assigned through a centralized randomization service in permuted blocks to one of four treatments”  Comment: Previous article (Vestbo 2016) has more detail |
| **Allocation concealment** *Selection bias* | Low | Quote: “Participants were randomly assigned through a centralized randomization service in permuted blocks to one of four treatments”  Comment: Previous article (Vestbo 2016) has more detail |
| **Selective reporting** *Reporting bias* | Low | All outcomes measures listed in methods section were reported in results |
| **Other sources of bias** *Other bias* | Low | No other sources of bias |
| **Blinding of participants and personnel** *Performance bias* | Low | Quote: “double-blind”  Comment: Probably done |
| **Blinding of outcome assessment** *Detection bias* | Low | Quote: “double-blind”  Comment: Probably done |
| **Incomplete outcome data** *Attrition bias* | Low | Quote: “were included in the intention-to-treat efﬁcacy population”; “Patients had to have a baseline measurement and at least one on-treatment measurement to be included in this analysis”  Comment: ITT population not always performed |

| **Author & year:** Vestbo 2016 | | |
| --- | --- | --- |
| **Domain** | **Risk of bias** | **Support for judgement** |
| **Random sequence generation**  *Selection bias* | Low | Quote: “The randomisation schedule was generated using the GSK validated randomisation software RANDALL.”  Comment: Probably done |
| **Allocation concealment** *Selection bias* | Low | Quote: “only the database administrators having knowledge of treatment assignment”  Comment: probably done |
| **Selective reporting** *Reporting bias* | Low | All outcomes measures listed in methods section were reported in results |
| **Other sources of bias** *Other bias* | Low | No other sources of bias |
| **Blinding of participants and personnel** *Performance bias* | Low | Quote: “double blind”  Comment: probably done |
| **Blinding of outcome assessment** *Detection bias* | Low | Quote: “double blind”  Comment: probably done |
| **Incomplete outcome data** *Attrition bias* | Low | Both “on-treatment” and “intention to treat” populations were used. |

| **Author & year:** Cazzola 2000 | | |
| --- | --- | --- |
| **Domain** | **Risk of bias** | **Support for judgement** |
| **Random sequence generation**  *Selection bias* | Low | Quote: “All patients who enter the run-in period were randomized to treatment in blocks of four according to a list of randomized codes”  Comment: Probably done |
| **Allocation concealment** *Selection bias* | Unclear | Quote: “randomized to treatment in blocks of four according to a list of randomized codes”  Comment: Unclear allocation concealment |
| **Selective reporting** *Reporting bias* | Low | All outcomes measures listed in methods section were reported in results |
| **Other sources of bias** *Other bias* | Low | No other sources of bias |
| **Blinding of participants and personnel** *Performance bias* | Unclear | Not mentioned. Only say “randomised. |
| **Blinding of outcome assessment** *Detection bias* | Unclear | Not mentioned. Only say “randomised”. |
| **Incomplete outcome data** *Attrition bias* | High | Quote: “In order to qualify for efficacy analysis, the patient had to complete the 3-month treatment period”; “69 patients completed the 3-month treatment period”  Comment: Complete case analysis. No mention of how many people were enrolled to start with. |

| **Author & year:** Vestbo 2005 | | |
| --- | --- | --- |
| **Domain** | **Risk of bias** | **Support for judgement** |
| **Random sequence generation**  *Selection bias* | Low | Not mentioned in article however, refer to paper Calverley 2003:  Quote:” We used a randomisation schedule generated by the patient allocation for clinical trials (PACT) program to assign patients to study treatment groups.”  Comment: Probably done |
| **Allocation concealment** *Selection bias* | Low | Not mentioned in article however, refer to paper Calverley 2003:  Quote: “Every participating centre was supplied with a list of patient numbers (assigned to patients at their first visit) and a list of treatment numbers.”  Comment: probably done |
| **Selective reporting** *Reporting bias* | Low | All outcomes measures listed in methods section were reported in results |
| **Other sources of bias** *Other bias* | Low | No other bias |
| **Blinding of participants and personnel** *Performance bias* | Low | Quote: “double blind”; (From Calverley 2003):“Study drugs were labelled in a way to ensure that both the patient and the investigator were unaware of the allocated treatment”  Comment: Probably done |
| **Blinding of outcome assessment** *Detection bias* | Low | Quote: “double blind”  Comment: Probably done |
| **Incomplete outcome data** *Attrition bias* | High | 140/361 withdrew from placebo, 119/372 withdrew from sal, 108/374 withdrew from flut, 89/358 withdrew from combination.  Quote: “Drop outs in the first 2 weeks were excluded from analyses”. (75 withdrew in first 2 weeks)  Comment: Not ITT analysis |

| **Author & year:** Renkema 1996 | | |
| --- | --- | --- |
| **Domain** | **Risk of bias** | **Support for judgement** |
| **Random sequence generation**  *Selection bias* | Low | Quote: “patients were allocated blindly (by computerized randomization stratified for smoking)”  Comment: Probably done |
| **Allocation concealment** *Selection bias* | Low | Quote: “computerized randomisation”  Comment: Probably done |
| **Selective reporting** *Reporting bias* | Low | All outcomes measures listed in methods section were reported in results |
| **Other sources of bias** *Other bias* | Low | No other biases |
| **Blinding of participants and personnel** *Performance bias* | Low | Quote: “double-blind”  Comment: Probably done |
| **Blinding of outcome assessment** *Detection bias* | Low | Quote: “double-blind”  Comment: Probably done |
| **Incomplete outcome data** *Attrition bias* | Low | 2/21 withdrew from bud, 4/19 withdrew from bud+pred, 5/18 withdrew from placebo  Quote: “For the whole group (n=58)..”  Comment: All randomised patients included |

| **Author & year: Lee 2015** | | |
| --- | --- | --- |
| **Domain** | **Risk of bias** | **Support for judgement** |
| **Random sequence generation**  *Selection bias* | Unclear | Not mentioned |
| **Allocation concealment** *Selection bias* | Unclear | Not mentioned |
| **Selective reporting** *Reporting bias* | Low | All outcomes measures listed in methods section were reported in results |
| **Other sources of bias** *Other bias* | Low | No other biases |
| **Blinding of participants and personnel** *Performance bias* | High | Quote: “Patients were randomised..”  Comment: No mention of blinding participants or personnel |
| **Blinding of outcome assessment** *Detection bias* | High | Quote: “Patients were randomised..”  Comment: No mention of blinding participants or personnel |
| **Incomplete outcome data** *Attrition bias* | Low | Quote: “FAS, full analysis set; SAS, safety analysis set”  Comment: lung function analysed in full analysis set ie all patients who were randomised |

| **Author & year: Shaker 2009** | | |
| --- | --- | --- |
| **Domain** | **Risk of bias** | **Support for judgement** |
| **Random sequence generation**  *Selection bias* | Low | Quote: “Patients were allocated into either group in a proportion of 1:1 by block randomisation using a random sequence generated by a computer program at AstraZeneca”  Comment: probably done |
| **Allocation concealment** *Selection bias* | Low | Quote: “Patients were allocated into either group in a proportion of 1:1 by block randomisation using a random sequence generated by a computer program at AstraZeneca”  Comment: probably done |
| **Selective reporting** *Reporting bias* | Low | All outcomes measures listed in methods section were reported in results |
| **Other sources of bias** *Other bias* | Low | No other biases |
| **Blinding of participants and personnel** *Performance bias* | Low | Quote: “double blinded” |
| **Blinding of outcome assessment** *Detection bias* | Low | Quote: “double blinded” |
| **Incomplete outcome data** *Attrition bias* | Unclear | 62/127 withdrew from placebo, 55/127 withdrew from ICS  Comment: Reported numbers are complete case for baseline characteristics however, random effects linear regression was used, which indicated complete case? |

| **Author & year: Wise 2000** | | |
| --- | --- | --- |
| **Domain** | **Risk of bias** | **Support for judgement** |
| **Random sequence generation**  *Selection bias* | Low | Quote: “The participants were randomly assigned to one of two treatment groups with stratification according to clinical centre and smoking status (participants were current smokers or had recently quit”  Comment: Probably done. Patients at individual test centres probably similar. |
| **Allocation concealment** *Selection bias* | Unclear | Not mentioned |
| **Selective reporting** *Reporting bias* | Low | All outcomes measures listed in methods section were reported in results |
| **Other sources of bias** *Other bias* | Low | No other biases |
| **Blinding of participants and personnel** *Performance bias* | Low | Quote: “The participants and clinical center staff were unaware of the study drug assignments” |
| **Blinding of outcome assessment** *Detection bias* | Low | Quote: “The participants and clinical center staff were unaware of the study drug assignments” |
| **Incomplete outcome data** *Attrition bias* | High | Quote: “The data were fitted to a linear longitudinal random-effects model”  38 withdrew from placebo, 28 withdrew from ICS  Comment: Complete case analysis used |

| **Author & year: Weir 1999** | | |
| --- | --- | --- |
| **Domain** | **Risk of bias** | **Support for judgement** |
| **Random sequence generation**  *Selection bias* | Unclear | Not mentioned |
| **Allocation concealment** *Selection bias* | Unclear | Not mentioned |
| **Selective reporting** *Reporting bias* | Low | All outcomes measures listed in methods section were reported in results |
| **Other sources of bias** *Other bias* | Low | No other biases |
| **Blinding of participants and personnel** *Performance bias* | Low | Quote: “double blind” |
| **Blinding of outcome assessment** *Detection bias* | Low | Quote: “double blind” |
| **Incomplete outcome data** *Attrition bias* | High | Quote: “Only patients with data points for at least 12 months were included in the final analysis”  Comment: Not all randomised patients included in analysis |

**References**

1. Collaborators GCoD. Global, regional, and national age-sex specific mortality for 264 causes of death, 1980-2016: a systematic analysis for the Global Burden of Disease Study 2016. Lancet 2017;390(10100).

2. Vogelmeier CF, Criner GJ, Martinez FJ, Anzueto A, Barnes PJ, Bourbeau J, et al. Global Strategy for the Diagnosis, Management, and Prevention of Chronic Obstructive Lung Disease 2017 Report. GOLD Executive Summary. Am J Respir Crit Care Med. 2017;195(5):557-82.

3. Fletcher C, Peto R. The natural history of chronic airflow obstruction. British Medical Journal 1977;1:1645-8.

4. Vestbo J, Anderson JA, Brook RD, Calverley PMA, Celli BR, Crim C, et al. Fluticasone furoate and vilanterol and survival in chronic obstructive pulmonary disease with heightened cardiovascular risk (SUMMIT): a double-blind randomised controlled trial. The Lancet. 2016;387(10030):1817-26.

5. Sutherland ER, Allmers H, Ayas NT, Venn AJ, Martin RJ. Inhaled corticosteroids reduce the progression of airflow limitation in chronic obstructive pulmonary disease: a metaanalysis. Thorax. 2003;58:937-41

6. Lapperre TS, Snoeck-Stroband JB, Gosman MME, Jansen DF, van Schadewljk A, Thladens HA, et al. Effect of Fluticasone With and Without Salmeterol on Pulmonary Outcomes in Chronic Obstructive Pulmonary Disease. Annals of Internal Medicine. 2009;151:517-27.

7. Calverley PM, Anderson JA, Brook RD, Crim C, Gallot N, Kilbride S, et al. Fluticasone furoate, vilanterol and lung function decline in patients with moderate COPD and heightened cardiovascular risk. American Journal of Respiratory and Critical Care Medicine. 2017.

8. Vestbo J, Edwards LD, Scanlon PD, Yates JC, Agusti A, Bakke P, et al. Changes in Forced Expiratory Volume in 1 Second over Time in COPD. The New England Journal of Medicine 2011;365(13):1184-92.

9. Tashkin DP. Variations in FEV(1) decline over time in chronic obstructive pulmonary disease and its implications. Curr Opin Pulm Med. 2013;19(2):116-24.

10. Woodcock A, Boucot I, Leather DA, Crawford J, Collier S, Bakerly ND, et al. Effectiveness versus efficacy trials in COPD: how study design influences outcomes and applicability. Eur Respir J. 2018;51(2).

11. Moher D, Shamseer L, Clarke M, Davina G, Liberati A, Petticrew M, et al. Preferred reporting items for systematic review and meta-analysis protocols (PRISMA-P) 2015 statement. Syst Rev. 2015;4(1):1.

12. Higgins J, Thompson SG, Deeks JJ, Altman DA. Measuring inconsistency in meta-analyses. BMJ. 2003;327:557-60.

13. Krishnan JK, Martinez FJ. Lung function trajectories and chronic obstructive pulmonary disease: current understanding and knowledge gaps. Current opinion in pulmonary medicine. 2018;24(2):124-9.
